# Understanding cingulotomy’s therapeutic effect in OCD through computer models

**DOI:** 10.3389/fnint.2022.889831

**Published:** 2023-01-10

**Authors:** Mohamed A. Sherif, Aryandokht Fotros, Benjamin D. Greenberg, Nicole C. R. McLaughlin

**Affiliations:** ^1^Department of Psychiatry, Brown University, Providence, RI, United States; ^2^Carney Institute for Brain Science, Brown University, Providence, RI, United States; ^3^Department of Psychiatry Lifespan Health System, Providence, RI, United States; ^4^Butler Hospital, Providence, RI, United States; ^5^United States Department of Veterans Affairs, Providence VA Medical Center, Providence, RI, United States

**Keywords:** cingulotomy, OCD, computer model, reinforcement learning, attractor dynamics

## Abstract

Cingulotomy is therapeutic in OCD, but what are the possible mechanisms? Computer models that formalize cortical OCD abnormalities and anterior cingulate cortex (ACC) function can help answer this. At the neural dynamics level, cortical dynamics in OCD have been modeled using attractor networks, where activity patterns resistant to change denote the inability to switch to new patterns, which can reflect inflexible thinking patterns or behaviors. From that perspective, cingulotomy might reduce the influence of difficult-to-escape ACC attractor dynamics on other cortical areas. At the functional level, computer formulations based on model-free reinforcement learning (RL) have been used to describe the multitude of phenomena ACC is involved in, such as tracking the timing of expected outcomes and estimating the cost of exerting cognitive control and effort. Different elements of model-free RL models of ACC could be affected by the inflexible cortical dynamics, making it challenging to update their values. An agent can also use a world model, a representation of how the states of the world change, to plan its actions, through model-based RL. OCD has been hypothesized to be driven by reduced certainty of how the brain’s world model describes changes. Cingulotomy might improve such uncertainties about the world and one’s actions, making it possible to trust the outcomes of these actions more and thus reduce the urge to collect more sensory information in the form of compulsions. Connecting the neural dynamics models with the functional formulations can provide new ways of understanding the role of ACC in OCD, with potential therapeutic insights.

## ACC lesion is therapeutic in OCD

The therapeutic effect of anterior cingulotomy in intractable OCD patients supports the involvement of the anterior cingulate cortex (ACC) in OCD pathophysiology. Anterior cingulotomy encompasses bilateral lesioning of Brodmann areas 24 and 32 and the underlying cingulum bundle. The first reports of cingulotomy go back to the 1940s when it was performed on patients with anxiety and chronic pain (Patel et al., 2013). In the mid-1960s, bilateral stereotactic cingulotomy was performed in patients with manic depressive symptoms. The study reported a significant improvement in 77% of patients with no significant complications (Ballantine et al., 1987). About 20 years later, the same authors reported a 56% improvement of OCD symptoms after anterior cingulotomy (Ballantine et al., 1987). Reanalysis of the treatment outcome with more rigid criteria suggested that the same data’s response rate is 33% (Cosgrove, 2000; Patel et al., 2013). Studies of anterior cingulotomy published in the following years reporting long-term follow-up of patients with OCD indicated a 32–48% response rate based on Y-BOCS scores (Dougherty et al., 2002; Jung et al., 2006).

Overall, anterior cingulotomy for OCD is recognized as a low-risk procedure. Dougherty et al. (2002) from Massachusetts General Hospital in Boston, USA, reported that one patient out of 44 experienced seizures that were controlled with anti-epileptics. Two patients described memory problems that were back to baseline within 6–12 months, and one patient described apathy with reduced energy that resolved after 6 months. Kim et al. (2003) reported no cognitive or physical side effects such as seizures after 3 months in 14 patients who underwent cingulotomies for OCD at Kwandong University in Kangnung, South Korea. This is unlike cingulotomy for intractable pain (usually due to malignancy) to reduce the distressing affective component of pain, where there have been reports of impaired self-initiated action (Cohen et al., 1999b) and sustained attention (Cohen et al., 1999a). Though there may be overlap in targeting, cingulotomy lesions for chronic pain are typically more caudal than those for OCD (Viswanathan et al., 2013). In OCD, studies did not show such impairments at 12 months follow up (Kim et al., 2003) or at a mean follow up duration of 32 months (Dougherty et al., 2002). Though no long-term side effects have been documented after cingulotomy for intractable OCD (Patel et al., 2013), more specific and sensitive (e.g., experimental cognitive) tasks might elucidate such changes.

Cingulotomy affects both gray and white matters. A simple extrapolation is that cingulotomy is therapeutic because it reduces the impact of functions and computations performed within the ACC on other cortical regions. ACC has been hypothesized to be involved in a multitude of functions, including monitoring task execution and errors, cognitive control, mapping stimuli and actions to outcomes of these actions, monitoring environment volatility, and decision making (Holroyd and Yeung, 2011). Understanding the computations of these functions can shed light on how cingulotomy might be helpful.

## Why computer models?

Computer models of a brain phenomenon fall along a spectrum. On one end are the models describing the neuronal dynamics in a particular brain region. On the other end are models of the different functions a region performs, reflected in behavioral patterns of the subjects. Between these two ends are models that perform computational functions implemented by elements constrained by neurobiology. This spectrum is analogous to the three levels of computations for analyzing cognitive processes proposed by Marr and Poggio (1976) and Marr (1982). Marr’s (1982) first level is the computations that need to take place in a brain region to solve a problem (computational level) (Barack and Krakauer, 2021). The second level is the steps needed to solve these computations (algorithmic level). These two levels correspond to models that describe the cognitive/psychological processes underlying a brain function and how they interact. The third level is the implementation of these steps (implementation or hardware level). This level corresponds to the neuronal models constrained by the neurobiology of the region(s) of interest and their dynamics.

Computational and mathematical modeling explicitly describe the mechanisms we hypothesize underlie a particular phenomenon, making these models falsifiable. Mathematically formulating the assumptions underlying our understanding makes computer models powerful tools in systematically navigating different hypotheses and mechanistic understandings of psychopathology (Volkow et al., 2013; Maia and Frank, 2017). We will discuss computer models of OCD neuronal dynamics and ACC functioning and speculate on possible bridges between them through cingulotomy. Such connections would be paramount in understanding the therapeutic effect as well as OCD psychopathology (Rolls, 2011).

## Neuronal models: Attractor dynamics

Attractor neural models simulate different dynamical states as neuronal firing patterns that are stable over time, called attractors. Brain states “jump” between these attractors, spending more time within an attractor rather than between attractors (Durstewitz et al., 2020). Attractor models have been used to represent the stability of information representation over time, e.g., in working memory (Verduzco-Flores et al., 2012).

Attractor models have also been used to represent how OCD patients appear behaviorally inflexible, stuck in specific thoughts (obsessions) or actions (compulsions). Rolls et al. (2008) hypothesized that cortical dynamics attractors in OCD have deep basins, making it difficult for the cortical dynamical states to “escape” from these attractors. For prefrontal regions involved in attention and planning, it will be difficult to switch attention when these regions are stuck in deep attractors. In the cingulate cortex, deep attractors will leave the patients at the mercy of fixated versions of ACC computations, as discussed in the next section. Rolls et al. (2008) model consisted of three interconnected populations of excitatory integrate-and-fire neurons: S1, S2, and NS (non-specific). The excitatory populations had AMPA and NMDA glutamatergic receptors, with strong recurrent connections within each neuronal population that supported persistent firing activity. The persistent firing was considered an attractor state. The excitatory neurons also received GABAergic input from a population of inhibitory interneurons. In response to input to either S1 or S2, excitatory neurons would go into persistent firing dynamics in the corresponding population, with a probability of 88%. Increasing conductance of NMDAR in the system by 3% raised the system’s probability of being in a persistent firing state to almost 100%. The probability also increased when the conductance of AMPAR was increased by 10%. The probability of staying in the persistent firing state was restored (the model became more flexible) by increasing GABA conductance by 10%. They also examined the model’s flexibility by introducing an input to one specific excitatory population while the other was firing. Increasing NMDAR conductance by 5% or AMPAR conductance by 10% made it more difficult for the model to respond to the second input (i.e., it became less distractible). The distractibility dynamics were rescued with GABAR augmentation.

As we will discuss later, the ACC is involved in high-level management of sequences of events or firing dynamics (Holroyd and Verguts, 2021), however, Rolls et al. (2008) model was of individual attractors. Maia and McClelland (2012) suggested using Verduzco-Flores et al. (2012) working memory model of *sequences* of attractors to reflect neuronal dynamics during varying excitation/inhibition balance in OCD (Maia and McClelland, 2012). Verduzco-Flores et al. (2012) model consisted of 80 excitatory units and 16 inhibitory units. When a sequence of activation of specific units was introduced, the model maintained its firing along the same sequence. When a new sequence was later introduced, the activation pattern switched to the new sequence. So, the model updated its activity after a new input. The model also had the feature of maintaining multiple inputs at the same time. But the model could not update the firing patterns after reducing the inhibitory influence to half the “control” value. The model either continued firing in the old pattern, or both patterns merged. Merging both patterns might reflect the elaboration and sophistication of compulsions. But such a reduction in inhibition is not reported in OCD microcircuit abnormalities. Maia and McClelland (2012) suggested that similar dynamics might arise with increased activation reported with hyperglutamatergic states (Pittenger et al., 2006).

To study the effect of glutamatergic and serotonergic changes, Maia and Cano-Colino (2015) developed an attractor model for orbitofrontal’s cortical dynamics. Similar cortical dynamics might apply to ACC, which also receives serotonergic input from the dorsal raphe nucleus (Chandler et al., 2013). The model investigated the role of tonic changes in serotonin levels, similar to what might happen with serotonin-reuptake inhibitors. In their model, serotonin had a predominantly inhibitory effect. This effect was mediated by the inhibitory influence of 5-HT_1A_ receptors on pyramidal neurons. The inhibitory effect was also mediated by the excitatory effect of 5-HT_2A_ receptors on PFC interneurons. Of note, 5-HT_2A_ receptors have a stimulatory effect on PFC pyramidal neurons mediated through increased intracellular calcium levels (Raymond et al., 2006). The model consisted of 1024 excitatory neurons and 256 inhibitory neurons recurrently connected. The neurons were arranged in a circle, and they were less connected to each other the further they were from one another. They modeled the effect of serotonin on pyramidal neurons as the summation of current from three mechanisms:


$$I_{5-HT}=I_{K1A}+I_{KCa}+I_{Can}$$

*I*_*K1A*_ was a potassium current mediated by 5-HT_1A_ activity. *I*_*KCa*_ was a calcium-gated potassium channel, and *I*_*Can*_ was a calcium-modulated cation channel. 5-HT_2A_ receptors mediated the activity of both *I*_*KCa*_ and *I*_*Can*_. Serotonin’s effect on interneurons was modeled as an inhibitory effect of 5-HT_2A_ receptors on the leak hyperpolarizing current, increasing interneuronal excitability. The model manifested two stable dynamics, low-rate and high-rate states. Increased 5-HT activity increased the probability of transition of the model from the high-rate to the low-rate stable state. Following an input that activated specific excitatory neurons, these neurons continued to fire. In response to a second input, the model either switched the activity to the second input, maintained firing corresponding to the first input (perseverance), or lost memory of both inputs. They found that perseverance increased with a reduction in 5-HT activity. To examine the effect of learning, they then implemented Hebbian plasticity. The network learned specific activity patterns and tended to return to them, developing attractors. The reduction of 5-HT sped up the formation of even more robust attractors, characterized by higher firing rates. Increased glutamatergic synaptic strength made the development of these attractors even more pronounced. In a separate set of simulations, increased glutamatergic synaptic strength (both AMPA and NMDA receptors) resulted in similarly robust attractors. Increasing 5-HT activity normalized the formation of these attractors (made the model less likely to develop them). There was even less formation of obsession-like attractors with 5-HT_2A_ antagonists or 5-HT_1A_ agonism. In these simulations, there was no baseline abnormality in serotonin levels. The model illustrated how tonic serotonin counteracted increased glutamatergic effects, making it easier to transition between attractors and not get stuck in them.

The attractor models we discussed highlighted that increasing glutamatergic transmission deepened the attractor basin, reducing the system’s flexibility. This was reversed by increasing GABAR currents (Rolls et al., 2008). The models’ findings hint at the involvement of the glutamatergic (Pittenger et al., 2011) and GABAergic systems in OCD pathology. Of note, these changes are opposite to what has been reported in the OFC in OCD from post-mortem examination (Piantadosi et al., 2019), where there was a reduction in mRNA copies of proteins involved in excitatory transmission. A possible explanation is that OCD psychopathology starts as reduced inhibition, as suggested by the attractor models. Then, to maintain the regional excitation/inhibition ratio (D’Souza and Burkhalter, 2017), there is compensatory reduction in expression of proteins involved in excitatory transmission, reflected in the reduction of mRNA copies encoding for these proteins.

## What do the effects of cingulotomy mean for attractor dynamics models?

The attractor models of OCD described cortical dynamics in PFC regions like the orbitofrontal and anterior cingulate cortex as states with deep basins. The deep basins make it difficult for the models to update their firing following new inputs. Thus, they can model the difficulty patients experience distracting themselves from the obsessions or stopping the compulsions. Dynamics within the ACC might spread to other regions through synchronization of oscillations in the theta range, which are prominent in the ACC. Regions with theta oscillations in the same phase as ACC theta have higher probability of responding to spikes in the ACC (Narayanan et al., 2013; Verguts, 2017). The spread of these dynamics to other cortical regions would make it difficult for the other regions to escape from such deep basins of persistent activity as well. Studies on either structural or functional connectivity post-cingulotomy for OCD are limited. However, Banks et al. (2015) evaluated presurgical structural connectivity using diffusion tensor imaging to identify predictors of response in 8 responders out of 15 patients. They found that pre-surgical increased right-sided structural connectivity between the lesion region and basal ganglia, hippocampus, and thalamus, predicted improvement in OCD symptoms, supporting the idea that cingulotomy might help reduce the influence of such abnormal dynamics on other cortical regions. Turning to functional models of the ACC, we will explore the implications of such limited flexibility.

## Functional models: Reinforcement learning (RL)

Neuronal firing dynamics in OCD have been modeled as attractors with deep basins, making it difficult to change the firing patterns, reflecting being stuck in behavior patterns. However, such firing dynamics can also reflect erroneous computations that control cognitive or motor behavior. To investigate this, we will look at ACC computations that affect behavior.

The ACC is involved in reward-guided learning and decision-making. ACC connections to the motor system suggest its ability to influence and be influenced by action selection. Lesion studies in monkeys show that ACC is more involved in action-reward associations rather than stimulus-reward associations like the lateral OFC (Rushworth et al., 2011). Reinforcement learning (RL) is a general computational framework that models behavior guided by action and reward. RL describes how an agent interacts with the environment and gets rewards. The goal of the agent is to maximize the amount of reward it gets. Predicting the rewards following an action and then comparing the prediction to the actual rewards it collected generates a reward prediction error. The agent then uses the prediction error to update its predictions. This is usually formalized as the “delta-rule” (Rescorla and Wagner, 1972; Collins and Shenhav, 2021):


V(t+1)=V(t)+alpha×predictionError

where V(t + 1) is the predicted reward value at time step t + 1, V(t) is the actual reward value obtained at the previous time step t, and alpha is a learning rate.

**FIGURE 1:**
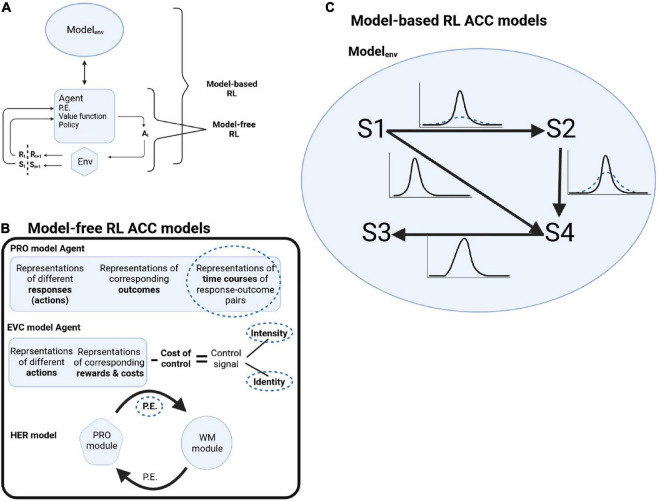
**(A)** Schematic of RL decision cycle. Following an action A_t_ chosen by the agent, the agent receives a reward R_t+1_ and the updated state of the environment S_t+1_. Model-free RL is when the agent is making decisions without a model of the world, while model-based RL is when the agent is using a model of the environment to plan its actions. **(B)** Schematic of model-free RL ACC models and where deep attractor dynamics might underline OCD pathology (dotted circles). P.E.: prediction error. **(C)** In model-based RL, the model of the environment is formalized as transition probabilities between states S1 to S4. In OCD, there is less confidence about the transition probabilities (dotted distributions).

Reinforcement learning consists of four elements (Sutton and Barto, 2018). After an agent acts on the environment, it gets information about the state of the environment after the action. The first element is the *reward signal* the agent gets from the environment at every time step. But agents are interested in the long-term rewards they can collect over many time steps, formulated as the *value function* (second element). The value function is calculated as the prediction of rewards given the environment’s state if the agent chooses its actions following a particular *policy* (third element). The agent might have a model of the environment (fourth element, which is optional), formalized as the probabilities of how the different states of the environment evolve to each other. When an agent is using such a model of the world to plan its actions, it is called model-based RL. When an agent uses the reward signal, value function, and policy, without using a model of the world, it is called model-free RL (Figure 1A).

Anterior cingulate cortex has been thought to represent an actor in an actor-critic RL, a formulation of model-free RL (Holroyd and Yeung, 2011). An actor-critic RL separates the estimation of the value function and prediction error (critic) from selecting the action (actor). An ACC lesion study highlighted this role (Modirrousta and Fellows, 2008). In an Eriksen flanker task, healthy subjects were allowed to respond after making a mistake by another button click. Their response time for the second click was around 200 ms, much faster than the reaction time for performing the task itself (about 400 ms). The shorter response time to the second click implied they were preparing for the second button press even before realizing they made a mistake. In five patients with lesions converging onto dorsal ACC, patients took around 700 ms to press the second button, even longer than the first button. This suggested the involvement of the ACC in action preparation for the second button press. Sheth et al. (2012) also found that cingulotomy abolished how reaction times are modulated by recent experience, supporting the predictive role of dorsal ACC (dACC). This predictive capacity shortens the reaction time when cognitive demand is constant, but prolongs the reaction time when there is more interference in a task, allowing for slower response and thus, more opportunity to consider the decision being made, reducing errors.

## Insights into OCD from applying attractor models to model-free RL

Multiple computer models described ACC functions based on the model-free RL framework, where the model is learning mappings of stimulus and action to outcome while monitoring deviations from expectations (prediction errors) (Vassena et al., 2017). Abnormal attractor dynamics might affect different computational elements. We will describe a number of these models and highlight how deep attractor dynamics can result in OCD symptomology (Figure 1B).

The Predictions of Response and Outcomes (PRO) model (Alexander and Brown, 2011) proposed how the ACC monitors errors and switches between tasks. The PRO model calculates the *probability* of different predicted outcomes following an action and their *timing* based on current context and stimuli (e.g., walking into a room, flicking a light switch, and light flooding the room). When cues were pointing to multiple possibilities, more predictions were made—for example, walking into a room and seeing both a light switch and a window that can be opened to allow air in. Another example is the Stroop task, where the ink color and the word predict different actions. Because multiple cues triggered more predictions, there was more activity in the model. Once a prediction was met (no prediction error), the activity decreased. One conclusion from the model was that the ACC was not necessarily encoding conflict. It instead encoded the multiple action possibilities when presented with cues that triggered more than one possible plan for action.

In the PRO model, the time course of the generated prediction error encoded the expected time of the predicted outcomes. The model separated the prediction error into positive signal (unexpected occurrence) and negative signal (unexpected non-occurrence) components that modulated learning. When a stimulus was presented and a prediction made, the negative component anticipated the predicted event’s occurrence. The amplitude of the negative component increased gradually as the timing of such an expected occurrence approached. When the anticipated event happened, the positive component of the prediction error signal increased, and the negative component was suppressed. When the anticipated event did not occur (negative surprise or unexpected non-occurrence), the negative component reached its peak around the expected time of occurrence, then gradually decreased without an increase in the positive component amplitude.

The validity of models is strengthened when they replicate experimental findings. The negative component of the PRO model explained the relationship between an EEG feature called the N2 event-related potential (ERP) component, and the speed-accuracy tradeoff in flanker tasks. In these tasks, a stimulus is shown consisting of elements of a particular kind, e.g., letters or digits. The central element can either be the same (e.g., three letters) or different (e.g., a central digit flanked by two letters), denoting congruent or incongruent trial, respectively. Subjects have to respond by pressing different buttons for congruent and incongruent trials. The N2 ERP component is recorded when these stimuli are presented, and usually has a larger amplitude in incongruent trials. The N2 ERP amplitude is also usually bigger in trials where subjects are spending longer time, possibly reflecting increased cognitive demand (Yeung and Nieuwenhuis, 2009). In PRO, when the response on a particular trial is delayed, the negative component will have a longer anticipation time and reach a higher amplitude. Using the negative component, the PRO model accurately calculated the N2 ERP amplitude dynamics.

The PRO model’s prediction error differs from the traditional prediction error implementation in the nigrostriatal pathway, which signals a discrepancy between an expected reward and the actual reward. The PRO model prediction error is generated when there is a discrepancy in predicting action-response pairs. The PRO model also makes multiple future predictions and assigns a probability to each, predicting rewarding and aversive outcomes.

The attractor dynamics in OCD could be mapped to different components of the PRO model. For example, the deep basins of attraction can reflect predicted times of occurrence that do not get updated. To illustrate, after turning the key to lock a door, I expect to hear the lock. The negative prediction error component should decrease after I hear the lock, signaling the end of the task (door is locked). However, suppose the neuronal dynamics representing the time of occurrence are stuck and not updated. In that case, there will still be the sense that it has not been locked, requiring rechecking or unlocking and relocking the door, seeking a reduction in prediction error. Cingulotomy might decrease such an error signal.

Anterior cingulate cortex is involved in multiple aspects of cognitive control, including exercising it, calculating its cost, and conscious awareness of mental effort during cognitive control. Naccache et al. (2005) described a patient with an ACC lesion who could not report which trials of the Stroop task were more difficult than others, while healthy subjects could. Multiple models captured these different aspects of cognitive control. For example, Shenhav et al. (2013) developed a dACC model that framed its prediction function in a control theory framework. In this context, control is an optimization problem. It means the ability to predict the rewards gained when selecting actions through different policies that govern reward over longer durations, then selecting the policy that maximizes the amount of reward gained (thus, optimizing the policy). Their model took into account the evidence that ACC is involved in both action selection and allocation of cognitive control resources and that cognitive control is costly. They proposed that the dACC predicts the reward and cost of each task choice, as well as the intrinsic cost of exerting control, generating the Expected Value of Control (EVC). EVC is a control signal with two components: identity and intensity. The control signal identity corresponds to the task choices, while its intensity modulates the magnitude of cognitive control needed. A change in the task rules would require updating the identity of the signal. In contrast, within a task with the same rules, a conflict of the correct response with a more automatic response, would require modulating the intensity of the signal. For example, in the Stroop task, when a subject has to name the ink color instead of reading the written color, the signal intensity would increase to allocate more resources for cognitive control.

Shenhav et al. (2013) model proposed that the dACC tries to maximize EVC. EVC was calculated by summing the probability of all future states based on the control signal needed and the current state, multiplied by the future states’ value. The cost of the control signal was then subtracted:


$$\begin{array}{l} EVC=\sum (Probability\,(futureStates|signal,\,currentStates)\cdot \\ \;\;\;\;\;\;\;value\,(futureStates\text{))}-signalCost \end{array}$$

The value of a future state was calculated from the immediate reward of the future state and the estimated EVC of the predicted future state.

Again, different elements of the EVC algorithm could be formulated as deep basins of attractions in OCD, making it difficult to update the EVC. McGovern and Sheth (McGovern and Sheth, 2017) suggested that in OCD, the ACC sends “mis-specified” signals of cognitive control, where the incorrect *identity* of the EVC, or inability to update it, can result in an inability to switch away from behaviors that are deemed unhelpful, as in compulsions. Increased *intensity* of EVC can result in feelings that patients had to do something about a situation, even if they cannot, increasing personal responsibility. They also suggested that impaired fear extinction in OCD could be linked to miscalculated EVC. In healthy people, repeated presentation of a conditioned stimulus without an aversive outcome reduces the fear of the aversive outcome. However, fear could be maintained by an inability to reduce the intensity of EVC because of persistent dynamics.

Cingulotomy will reduce the interaction between the ACC and other brain regions. Alexander and Brown, 2015 expanded the PRO model to study interactions of medial PFC (ACC) with the dorsolateral PFC (DLPFC) in a model called the Hierarchical Error Representation (HER) model. In HER, the hierarchies of representations reflect the gradient of abstraction reported in the PFC [e.g., (Koechlin et al., 2003)]. Each level has a medial component [representing the ACC/medial PFC (mPFC)] that consists of a PRO module, and a lateral component (representing DLPFC) consists of a working memory module. The idea behind the model is that within each level, the medial component makes predictions about response and outcome and calculates prediction errors that are passed to the lateral component. The lateral component (working memory module) would maintain these prediction errors and modulate how the medial component makes the next prediction based on the sensory stimuli features maintained in the working memory module. So, prediction errors generated in the ACC are passed onto DLPFC to aid in its training, while prediction errors generated within the DLPFC module are passed to ACC to update its predictions of responses based on actions taken by the agent. The prediction error is used to update the predictions of the modules within a level. The prediction error is also passed to the next level of the hierarchy. So the higher (more abstract) levels generate predictions related to the errors they receive from the lower levels (thus, the name hierarchical error representation).

If the error calculating component of the mPFC/ACC in the HER model is stuck in an attractor, the DLPFC component will keep getting an error signal and updating its predictions. The predictions are sent back to the mPFC/ACC, which in turn keeps sending an error signal to the DLPFC component. This will be similar to turning a key to lock the door. Instead of receiving no error since the door has been locked, the DLPFC module will get an error from the mPFC signaling something is wrong with the prediction made by the DLPFC that the door is locked—so the door still needs to be locked! Cingulotomy in OCD might reduce such a constant error signal, allowing the DLFPC to leave the predictions it made unaltered.

The hierarchical nature of representation behind the HER model could be applied in a different way to RL (hierarchical RL). In their formulation, Holroyd and Verguts suggested that the ACC is not interacting with the environment by choosing single actions, but rather by choosing sequences of actions, called strategies or options, that are encapsulated in hierarchies (Holroyd and Verguts, 2021). For example, get gas and buy groceries instead of individual actions like pushing the gas pedal and turning the steering wheel to the right. Making decisions in a hierarchical manner, where low-level sequences are encapsulated together, allows focusing on choosing the needed high-level action without being distracted by the sequences at the lower level. The sophistication of compulsions and the urge to do them in specific ways suggest that the ACC sends information of hierarchical nature to stay on a particular goal. Such “fixed” goal representation could be mediated by a deep attractor basin, reducing the likelihood of escaping it, and thus making it difficult for the ACC to move onto other high-level goals. Cingulotomy might reduce the influence of such dynamics on regions involved in the execution of lower-level steps. An interesting finding in lesions of ACC is that it does not affect learning from feedback on a trial-by-trial basis, but rather higher-level changes like reduced spontaneity, supporting the hierarchical nature of RL implemented in the ACC (Holroyd and Yeung, 2012).

## Model-based RL, OCD, and attractor models

The above models were formulated through model-free RL, where an agent selects actions given the reward values predicted for each action, learned through interacting with the world. But an agent can also learn a model of the world while the agent is interacting with it, then uses the model to plan its future actions. Having a model of the environment is the optimal way to exert control (Conant and Ross Ashby, 1970), and animals seem to build world models when uncertainty about the environment is high (Monosov, 2020). In RL, this is called model-based RL. According to the Bayesian-brain theory (Knill and Pouget, 2004; Friston, 2010), the experienced world consists of time-evolving states hidden from the brain. To build a model of the world, the brain tries to infer such hidden states. The brain makes a current inference, which is then compared with the incoming perception. When there is a discrepancy, the brain generates a prediction error that is used to update the predicted future state, i.e., to update the model of the world. Note that the prediction error here is about the model of the world [called state prediction error (Gläscher et al., 2010)] rather than about the value of predicted reward as in model-free RL. Inferring the hidden states depends on (1) what is observed from perceptions and exploratory behavior and (2) assumptions about how the states evolve over time based on past experience (state transitions). The world model consists of the state transitions, as well as the confidence about them. For example, if I locked the door, what is the probability of the door staying locked, and how confident am I about this probability. State transitions either depend on actions taken by a person (action-dependent transitions) or are action-independent.

An OCD formulation based on learning a world model hypothesized that OCD results from uncertainty about the state transitions (Fradkin et al., 2020a). Fradkin et al. (2020a) suggested that reduced confidence in how the states will change underlies OCD. Patients will continue seeking ways to reduce the uncertainty, which can manifest as compulsions, as biological agents are biased toward seeking information to reduce uncertainty (Monosov, 2020). For example, if I turn the stove knob to OFF, and I can see that it is pointing to OFF, my prediction will be that it will stay in the OFF state, i.e., the transition to a not-OFF state will be pretty low, and I am confident about that. However, if my confidence in this transition probability (action-independent transition) is low, I would doubt whether it stayed off, and I will have an urge to look at it again to make sure. And if this is not enough, I would touch it to make sure it is in the off position. But then, if my confidence in how my actions can change objects (action-dependent transition) is low as well, I will keep touching it to ensure it stayed OFF. The lower transition certainty resulted in the increased weighting of prediction errors, urging me to collect more sensory information by checking.

Fradkin et al. (2020b) distinguish between state prediction errors related to the current state and future states. Prediction errors associated with the current state would manifest as internally generated obsessions or obsessions related to incompleteness. Compulsions will be directed at getting to the just-right experience (Rasmussen and Eisen, 1992; Summerfeldt, 2004), aiming to reduce the current prediction error by collecting more perceptual input, as in checking. Harm-avoidance obsessions and their compulsions are aimed at reducing prediction errors related to future states, thus minimizing the risk of future harm (Salkovskis, 1999). In both cases, reduced transition certainty would drive patients to perform the compulsions repeatedly. But because of reduced certainty in action-dependent transitions as well, performing the compulsions will increase uncertainty, driving more compulsions. To increase their confidence in action-dependent transitions, patients rely on habitual actions that they have learned by repetition. In a later study, Fradkin et al. found that subjects with high OC symptoms seem to rely less on the past (Fradkin et al., 2020b), supporting reduced confidence in how past states will evolve.

Cortical dynamics representing uncertainty [e.g., (Monosov, 2020)], stuck in attractors with deep basins can reflect a persistent transition uncertainty, i.e., difficulty in becoming more confident about transitions (Figure 1C). Reduced transition certainty would drive the prediction that the task outcome was undone, e.g., a locked door might not have stayed locked. ACC plays a role in selecting sequences of actions and in goal-directed behavior, monitoring the state of a task and its subgoals, and thus informing about switching a subgoal to the next subgoal. This can be through a sequence of attractors. In OCD, with deep attractors, patients get stuck in a subgoal, repeating it over and over again, and ACC signals that the subgoal is not complete yet. With cingulotomy, tissue removal might mean removing parts of the neuronal ensembles participating in the attractors, thus making them less resistant to change, reducing their depth and making it easier not to be stuck in them. This might reduce such a signal delivered to other brain regions involved in completing the task, reducing the persistence of obsessions or the urge of compulsions.

## Discussion

In this short review, we discussed potential mechanisms of the therapeutic effects of cingulotomy in OCD guided by computer models along a spectrum encompassing Marr’s three levels of analysis. On the neurobiological end are attractor models focused on neurobiologically plausible ways of conceptualizing obsessions and compulsions. On the functional end, models formalized how the ACC executes its multitude of functions without necessarily discussing their neurobiological implementation.

The brain, however, occupies both ends of the spectrum. It performs complex functions using biological elements. Thus, our discussion tried to bridge models across the spectrum. For example, assuming that hyperactive ACC might reflect deep attractors, these attractors might represent persistent incorrect predictions difficult for patients to escape. The attractors might also represent neuronal states constantly generating prediction errors. Another possibility is that the persistent cortical dynamics underlie transition uncertainty, driving patients to seek more perceptual input as they have low confidence in their predictions. In general, cingulotomy might reduce the size and amount of pathological neuronal ensembles stuck in deep attractors in the dACC that is constantly sending signals of such persistent dynamics on other brain regions. This would result in less influence of these ensembles that might be reflected in the lower amplitude of such signals or lower entrainment of other regions to these signals. When we seek to understand interventions done on the “brain hardware,” constraining the functional level models by biology will be an essential research direction (Niv and Langdon, 2016).

We focused on computations happening locally within the ACC. Because of the wide connectivity of ACC, the implications of these local computations could be widespread. Although early neurocircuitry diagrams of OCD have delineated frontal-subcortical pathways as implicated in symptom presentation, newer models [e.g., (Shephard et al., 2021)], have detailed the likely complexity of the connectivity patterns. The ACC is likely a “hub” in several of the circuits (e.g., fronto-subcortical, sensorimotor circuits) involved in variable presentations of OCD. These differing circuits may have relevance to individualizing neurocircuitry-based treatments, as OCD subtypes may relate to disparate circuitry. Though not entirely clear, there are early suggestions that there may be neurocircuitry differences between various OCD symptom presentations. For example, given that the incompleteness (“just right”) subtype of OCD is dominated by the feeling of lack of completion, or the continued presence of an error signal, the ACC may be especially relevant for those with these symptoms. Though research is not clear thus far, it is possible that different ablative targets (e.g., cingulotomy vs. capsulotomy) are more effective for particular symptom presentation. In addition, given that these constructs discussed above are relevant for the functioning of the anterior cingulate in general, targeting the ACC may be effective not only for targeting OCD but also for other disorders.

To test the predictions we presented here, we need electrophysiological recordings from the ACC and its target regions while patients perform tasks, both before and after cingulotomy. There is a growing interest in understanding neuronal firing as population dynamics, including attractors (Ebitz and Hayden, 2021). Some work has been done on neuronal dynamics of the subthalamic nucleus, e.g., (Burbaud et al., 2013), but little on *neocortical* dynamics in OCD. Connecting neuronal dynamics of ACC in OCD to behavior will allow us to delineate which of the functional models agree more with electrophysiology. For example, we can study prediction error computation and how it correlates with spikes, local field potentials, or both. Connecting ACC dynamics to aspects like flawed prediction errors would then make it possible to identify patients with a higher chance of improvement following cingulotomy.

## Author contributions

MS wrote the first draft of the manuscript. AF and NM wrote sections of the manuscript. All authors contributed to the manuscript revision, read, and approved the submitted version.
